# D3EGFR: a webserver for deep learning-guided drug sensitivity prediction and drug response information retrieval for EGFR mutation-driven lung cancer

**DOI:** 10.1093/bib/bbae121

**Published:** 2024-03-28

**Authors:** Yulong Shi, Chongwu Li, Xinben Zhang, Cheng Peng, Peng Sun, Qian Zhang, Leilei Wu, Ying Ding, Dong Xie, Zhijian Xu, Weiliang Zhu

**Affiliations:** State Key Laboratory of Drug Research; Drug Discovery and Design Center, Shanghai Institute of Materia Medica, Chinese Academy of Sciences, Shanghai 201203, China; School of Pharmacy, University of Chinese Academy of Sciences, Beijing 100049, China; Department of Thoracic Surgery, Shanghai Pulmonary Hospital, Tongji University School of Medicine, Shanghai 200433, China; State Key Laboratory of Drug Research; Drug Discovery and Design Center, Shanghai Institute of Materia Medica, Chinese Academy of Sciences, Shanghai 201203, China; State Key Laboratory of Drug Research; Drug Discovery and Design Center, Shanghai Institute of Materia Medica, Chinese Academy of Sciences, Shanghai 201203, China; School of Pharmacy, University of Chinese Academy of Sciences, Beijing 100049, China; Key Laboratory of Human Functional Genomics of Jiangsu Province, Department of Biochemistry and Molecular Biology, Nanjing Medical University, Nanjing 211166, China; School of Computer Science and Technology, East China Normal University, Shanghai 200062, China; Department of Thoracic Surgery, Shanghai Pulmonary Hospital, Tongji University School of Medicine, Shanghai 200433, China; Department of Pathology, the First Affiliated Hospital of Nanjing Medical University, Nanjing 210029, China; Department of Thoracic Surgery, Shanghai Pulmonary Hospital, Tongji University School of Medicine, Shanghai 200433, China; State Key Laboratory of Drug Research; Drug Discovery and Design Center, Shanghai Institute of Materia Medica, Chinese Academy of Sciences, Shanghai 201203, China; School of Pharmacy, University of Chinese Academy of Sciences, Beijing 100049, China; State Key Laboratory of Drug Research; Drug Discovery and Design Center, Shanghai Institute of Materia Medica, Chinese Academy of Sciences, Shanghai 201203, China; School of Pharmacy, University of Chinese Academy of Sciences, Beijing 100049, China

**Keywords:** lung cancer, EGFR mutation, drug sensitivity prediction, patient case database, deep learning

## Abstract

As key oncogenic drivers in non-small-cell lung cancer (NSCLC), various mutations in the epidermal growth factor receptor (EGFR) with variable drug sensitivities have been a major obstacle for precision medicine. To achieve clinical-level drug recommendations, a platform for clinical patient case retrieval and reliable drug sensitivity prediction is highly expected. Therefore, we built a database, D3EGFRdb, with the clinicopathologic characteristics and drug responses of 1339 patients with EGFR mutations via literature mining. On the basis of D3EGFRdb, we developed a deep learning-based prediction model, D3EGFRAI, for drug sensitivity prediction of new EGFR mutation-driven NSCLC. Model validations of D3EGFRAI showed a prediction accuracy of 0.81 and 0.85 for patients from D3EGFRdb and our hospitals, respectively. Furthermore, mutation scanning of the crucial residues inside drug-binding pockets, which may occur in the future, was performed to explore their drug sensitivity changes. D3EGFR is the first platform to achieve clinical-level drug response prediction of all approved small molecule drugs for EGFR mutation-driven lung cancer and is freely accessible at https://www.d3pharma.com/D3EGFR/index.php.

## INTRODUCTION

Lung cancer is the most common malignant disease and the leading cause of cancer mortality worldwide, causing approximately 2.2 million new cases and 1.8 million deaths in 2020 [1]. Non-small-cell lung cancer (NSCLC) accounts for 85% of all lung malignancies [2, 3], mainly comprising adenocarcinoma (ADC), squamous cell carcinoma and large cell carcinoma. Epidermal growth factor receptor (EGFR) mutations are closely associated with carcinogenesis [4], and have been identified in approximately 32.3% of NSCLC [5]. Mutations in the kinase domain of EGFR can promote ligand-independent dimerization and activation of the receptor, resulting in constitutive activation of downstream signaling pathways to induce tumorigenesis [6, 7].

EGFR–tyrosine kinase inhibitors (EGFR-TKIs) are used as the standard treatment for patients with advanced EGFR mutation-driven lung cancer [8, 9]. In patients with EGFR-sensitive mutations, compared with platinum-based chemotherapy, EGFR–TKIs significantly improve the objective response rate and prolong progression-free survival (PFS) and overall survival (OS) rates [10–12]. However, patients with different EGFR mutations exhibit varying responses to EGFR–TKIs, mainly because of either intrinsic or acquired resistance [13]. The development of DNA sequencing technologies has enabled the identification of several novel and uncharacterized EGFR variants [14], which makes precision medicine more challenging for patients with new mutations [15, 16].

To date, only nine small-molecule drugs have been approved for the treatment of patients with metastatic EGFR mutation-positive NSCLC worldwide (Table S1). The first-generation EGFR–TKIs, gefitinib and erlotinib, have been used as the first-line therapy for patients with common EGFR mutations such as exon 19 deletion (19del) or L858R point mutation [17, 18], and the third-generation agent, osimertinib, could benefit patients with the T790M resistant mutation [19, 20]. However, the efficacy of these EGFR–TKIs in the treatment of patients with uncommon or new EGFR mutations remains inadequately elucidated.

Profiting from the cumulative experience of relevant clinical trials over the past two decades, the risks of adverse effects and poor therapeutic efficacy in patients with common mutations could remain low throughout the entire treatment period. Noteworthy, the individual characteristics of patients, including gender, age and smoking status, are also related to the incidence rate of EGFR mutation-driven lung cancer [5, 21]. Although considerable progress has been made in integrating information on EGFR mutants and targeted drugs [22–27], systematic retrospective clinical analysis has been limited by the absence of credible resources profiling the clinical characteristics and outcomes of patients with EGFR mutations. Thus, a comprehensive and searchable database with details of patient cases, including EGFR mutation, clinicopathological characteristics and therapeutic response of approved drugs, is highly needed for making treatment decisions.

In addition, for rare or newly emerged variants, the EGFR mutation status has been attempted as a predictive and prognostic marker for predicting the effect of targeted therapy [28–30]. For instance, Ikemura *et al*. [31] successfully predicted the diverse *in vitro* and *in vivo* sensitivities of exon 20 insertion mutants using molecular dynamics (MD) simulations, in which the Δ*G*_bind_ value for a certain mutant–inhibitor complex can be obtained in approximately 1 week. Wang *et al*. [32] combined MD and extreme learning machines to construct a personalized drug resistance prediction model. However, the limitations of high time consumption and the computational costs of MD simulations obstruct their wide application. In addition, the previous studies only predict drug response for two or fewer drugs (Table S2). Recently, artificial intelligence has shown increased expressive power in identifying, processing and extrapolating drug-target interactions based on existing biological activity data [33, 34], which could be an effective tool for developing a fast and accurate drug sensitivity prediction model for rare and newly emerged mutations.

In this study, we aimed to investigate the impact of EGFR mutations on drug sensitivity and provide optimal treatment guidance through a real patient case database and a drug sensitivity prediction tool. First, the overall information on the D3EGFRdb clinical patient database was introduced, including the number of literature sources and cases, the distribution of individual patient characteristics and the analysis of statistical results. Second, the feasibility of molecular docking and deep learning approaches in predicting drug sensitivity was evaluated and the selected deep learning model was used to explore potential changes in drug sensitivity caused by amino acid mutations around the drug-binding pocket of EGFR. Finally, the construction and usage of the D3EGFR website were introduced to assist users in effectively using the D3EGFRdb patient database and D3EGFRAI prediction model.

## MATERIALS AND METHODS

### Construction of a clinical medication database for patients with EGFR mutations

A literature search was performed in PubMed [35] for relevant studies published before 16 February, 2023. The specific search strategy was as follows: (i) the title or abstract of the literature must contain the keywords ‘EGFR mutation’ and ‘non-small cell lung cancer’, (ii) the title or abstract of the literature must contain at least one approved EGFR–TKI agent, including ‘tyrosine kinase inhibitors’, ‘gefitinib’, ‘erlotinib’, ‘icotinib’, ‘afatinib’, ‘osimertinib’, ‘olmutinib’, ‘dacomitinib’, ‘almonertinib’ and ‘furmonertinib’ and (iii) the full text of the literature must contain the keywords about drug responses. Drug response is evaluated based on the World Health Organization criteria [36] and Response Evaluation Criteria in Solid Tumours (RECIST) V1.0 or V1.1 guidelines [37, 38], which are divided into complete response (CR), partial response (PR), stable disease (SD) and progressive disease (PD). Therefore, the full text of the literature must contain at least one of the following four keywords: ‘complete response’, ‘partial response’, ‘stable disease’ and ‘progressive disease’.

### Prediction of the drug sensitivity of EGFR mutants based on molecular docking

Molecular docking is a structure-based strategy for predicting potential binding between a drug and a protein [39]. Using this strategy, various docking models were constructed for different mutated EGFRs. The correlation between the docking score and bioactivity was then calculated to analyse the feasibility of drug sensitivity prediction of EGFR mutants. The bioactivity dataset used in this study is from the report by Robichaux *et al*. [40], covering 1349 experimentally measured biological activities (log(mutant/wild type) of IC_50_ values) of 18 EGFR-TKIs and 77 EGFR mutants. After excluding mutants that only report mutant exons, such as 19del, we performed homology modeling to construct 3D structures for 64 mutants with clear mutation sites using the X-ray structure (PDB id: 3POZ, Resolution: 1.50 Å) as the template using MODELLER (version 9.24) [41]. The generated mutant protein structures were protonated at pH 7.4 using pdb2pqr software [42]. Molecular docking was performed using smina [43], which is a fork of AutoDock Vina [44] with improved docking performance. The docking boxes of all mutants were generated by extending 4 Å in each dimension based on the coordinates of the reference ligand in the crystal complex. Docking was performed using random seed 0.

### Deep learning model for predicting the drug sensitivity of EGFR mutations

Given that deep learning can perform feature detection from large-scale bioactivity data and has flexible neural network architectures, it has achieved remarkable success in the prediction of drug-target interactions [45]. In addition, deep learning can be independent of the 3D structures of proteins, thereby avoiding biases caused by structural modeling. In this study, we explored deep learning models with different encoder combinations for drugs and protein mutants to identify the optimal model for drug sensitivity prediction. The drug and protein encoders were provided by DeepPurpose [46], with 80 encoder combinations (Table 1). Regarding datasets, 1/10 of the 1349 experimentally determined biological activity data were taken as the test set and the remaining data were further randomly divided into 10 different training and validation sets at a ratio of 9:1 for 10-fold cross-validation. Models with an average Pearson correlation of 10-fold higher than 0.8 on the validation set were retained. The formulas of the related evaluation metrics are as follows:


(1) \begin{equation*} \mathrm{Pearson}\ \mathrm{correlation}=\frac{\mathrm{N}\sum{x}_i{y}_i-\sum{x}_i\sum{y}_i}{\sqrt{\mathrm{N}\sum{x}_i^2-{\left(\sum{x}_i\right)}^2}\sqrt{\mathrm{N}\sum{y}_i^2-{\left(\sum{y}_i\right)}^2}} \end{equation*}

(2) \begin{equation*} \mathrm{Mean}\ \mathrm{Square}\ \mathrm{error}\ \left(\mathrm{MSE}\right)=\frac{1}{N}\sum_{i=1}^n{\left({x}_i-{y}_i\right)}^2 \end{equation*}

where N represents the number of samples, while ${x}_i$ and ${y}_i$ represent the labels and predicted values of the samples, respectively.

**Table 1:** Encoders for drugs and protein mutants

Type	Encoder
Drug	CNN, CNN_RNN, Daylight, ErG, ESPF, Morgan, MPNN, Pubchem, rdkit_2d_normalized, Transformer
Mutant	AAC, CNN, CNN_RNN, Conjoint_triad, ESPF, PseudoAAC, Quasi-seq, Transformer

**Figure 1:**
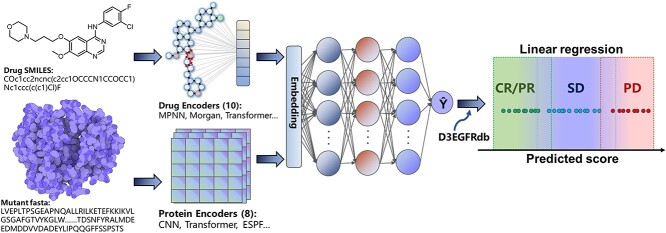
Frameworks of prediction models using different encoder combinations for drugs and protein mutants.

Then, the test set was used to evaluate the retrained models by merging the training and validation sets. Subsequently, we predicted the binding affinity of mutations collected in D3EGFRdb and mapped the predicted value with the drug response using a multinomial logistic regression analysis, which was taken from the sklearn machine learning library. For the multinomial logistic regression analysis, we used the solver of ‘newton-cg’, penalty of ‘l2’, C of 1.0, as well as the balanced mode to automatically adjust weights inversely proportional to class frequencies in the input data. Figure 1 illustrates the framework of the drug sensitivity prediction model D3EGFRAI.

### Average clinical drug response (ACR) for the quantitative representation of drug response

Because of the influence of individual differences and other complex factors, patients with the same mutation type and the same drug administration may have different drug responses. For instance, in D3EGFRdb, five patients with the D770insSVD mutation were treated with erlotinib, among whom three showed PD response and the other two showed SD response, suggesting an unreasonable direct adaptation of individual labels for model evaluation. Thus, we defined an ACR to represent the overall efficacy of patients with the same mutation type and drug treatment. Drug responses were converted to numerical values such that CR/PR = −1, SD = 0 and PD = 1. Then, the same drug–mutant cases with some patient cases greater than 3 in D3EGFRdb were screened and their average clinical response value (ACRV) was calculated using equation 3 and was further converted to ACR using equation 4. Thus, we constructed a representative D3EGFRdb subset with 43 drug–mutant pairs for model evaluation.


(3) \begin{equation*} \mathrm{ACRV}=\frac{\left(-1\right)\times{\mathrm{N}}_{\mathrm{CR}/\mathrm{PR}}+0\times{\mathrm{N}}_{\mathrm{SD}}+1\times{\mathrm{N}}_{\mathrm{PD}}}{{\mathrm{N}}_{\mathrm{CR}}+{\mathrm{N}}_{\mathrm{PR}}+{\mathrm{N}}_{\mathrm{SD}}+{\mathrm{N}}_{\mathrm{PD}}} \end{equation*}

(4) \begin{equation*} {\displaystyle \begin{array}{c}\mathrm{ACR}=\left\{\begin{array}{c}\mathrm{PD}\ \mathrm{ACR}\mathrm{V}>0.5\\{}\mathrm{SD}\ 0.5\ge \mathrm{ACRV}>-0.5\\{}\mathrm{CR}/\mathrm{PR}\ \mathrm{ACR}\mathrm{V}\le -0.5\end{array}\right.\ \end{array}} \end{equation*}

where N_CR/PR_, N_SD_ and N_PD_ are the numbers of CR/PR, SD and PD patients with the same mutation type and drug treatment, respectively.

## RESULTS

### D3EGFRdb overview and statistical analysis

Through systematic literature search and manual collation, 141 studies on the clinical medication and drug responses of patients with EGFR mutations were identified, of which 108 were retrospective case reports/series, 26 were prospective clinical trials and 7 were prospective cohort studies. All patients with EGFR mutations were collected and annotated with clinical information (such as mutation site, gender, age, smoking status, pathology and EGFR–TKI treatment), clinical outcomes (such as drug response, time to progression, PFS and OS), study type and original literature for convenience. Based on this information, we constructed a clinical medication database D3EGFRdb for patients with EGFR mutations. D3EGFRdb contained a total of 1339 patients with 257 different mutation types, including 1032 patients in the response group (CR/PR/SD) and 307 patients in the non-response group (PD).

**Figure 2:**
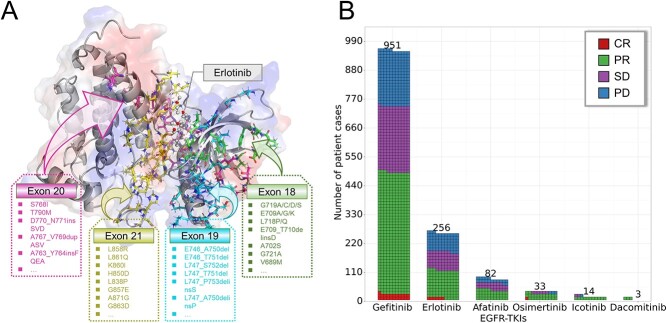
Mutation status and clinical outcomes of patients in D3EGFRdb. (**A**) Distribution of hotspot mutations. (**B**) Distribution of patient cases with different drug responses to EGFR-TKIs. A grid point represents a case.

The reported mutation sites were mainly located in exons 18–21 (Figure 2**A**), which encode the tyrosine kinase domain of the *EGFR* gene and are the binding sites of available drugs. For instance, exon 19 deletion and exon 21 L858R are the most common *EGFR* mutations in these regions, whereas less common mutations include G719X and E709X in exon 18, S768I and T790M in exon 20 and L861Q and K860I in exon 21. Bringing a comparative perspective to the clinical application of EGFR–TKIs, the first-generation inhibitor gefitinib from AstraZeneca is the most extensively used and widely studied EGFR–TKI (951 cases, 71.0%), followed by another first-generation inhibitor erlotinib (256 cases, 19.1%). Gefitinib was found to be slightly better than erlotinib in terms of clinical drug response (gefitinib, CR/PR versus SD/PD: 51.2% versus 48.8%; erlotinib, CR/PR versus SD/PD: 44.1% versus 55.9%) (Figure 2**B**). The relatively low use of the second-generation inhibitors afatinib and dacomitinib is associated with increased toxicity through non-specific targeting of wild-type EGFR [47, 48]. The third-generation EGFR–TKI osimertinib is the first FDA- and EMA-approved EGFR–TKI for treating patients with metastatic NSCLC who have a T790M resistance mutation [49]. In addition, icotinib is a potent and specific EGFR–TKI that was approved in China in 2011 [50]. The above information together with gender, age, smoking status, pathology, time to progression, PFS, OS, study type and original literature were collected in D3EGFRdb, making it a comprehensive database for retrospective medical records search.

**Figure 3:**
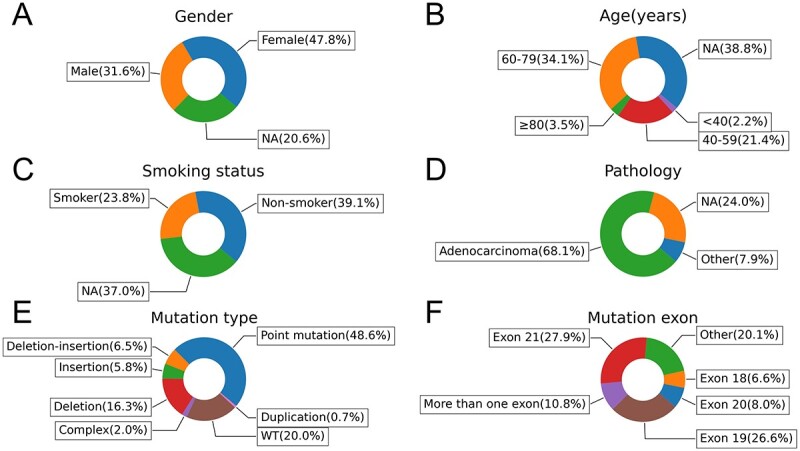
Pie charts of gender, age, smoking status, pathology, mutation type and mutant exon of patients in D3EGFRdb.

Multivariate analysis of D3EGFRdb (Figure 3) revealed that females (females versus males: 47.8% versus 31.6%), individuals aged 60–79 years (34.1%) and non-smokers (non-smoker versus smoker: 39.1% versus 23.8%) were the most prevalent patients with EGFR mutations. This suggests that individual characteristics of patients are associated with the incidence of EGFR-mutant lung cancer, which is consistent with the findings of Zhang and Shigematsu [5, 21]. In addition, the predominant pathology was adenocarcinoma (ADC versus non-ADC: 68.1% versus 7.9%) in the reported patient series. Furthermore, point mutation is the most common mutation (48.6%), followed by deletion mutation (16.3%), mainly comprising the common L858R substitution in exon 21 and deletion mutations in exon 19.

### External clinical dataset for assessment

To validate the prediction model using D3EGFRAI, the clinical information and outcomes of 102 patients treated with EGFR-TKIs in the Shanghai Pulmonary Hospital between March 2015 and October 2020 were used as the external clinical dataset (Table 2 and Table S3). The Ethics Committee of Shanghai Pulmonary Hospital approved this study and informed consent was waived because this was a retrospective study. Their age range was 33–85 years with a median age of 61 years and their histology was mostly adenocarcinoma. Objective responses to EGFR–TKIs were evaluated according to the RECIST V1.1 guidelines [38]. There were 13 different types of drug–mutant information pairs (drug–mutant pair hereinafter) in these 102 patient cases and their average clinical drug response (ACR) was re-evaluated.

**Table 2:** Clinicopathological characteristics of patients in the external clinical dataset

Characteristic	No. of patients (N = 102)
Age (years)	
Median	61
Range	33–85
Gender	
Male	50 (49.0%)
Female	52 (51.0%)
Smoking status	
Current	3 (2.9%)
Former	28 (27.5%)
Never	71 (69.6%)
Histology	
Adenocarcinoma	100 (98.0%)
Squamous cell carcinoma	1 (1.0%)
Large cell carcinoma	1 (1.0%)
EGFR-TKIs	
Erlotinib	10 (9.8%)
Gefitinib	52 (51.0%)
Icotinib	34 (33.3%)
Osimertinib	5 (4.9%)
Afatinib	1 (1.0%)
Response to EGFR-TKIs	
Partial response	67 (65.7%)
Stable disease	31 (30.4%)
Progressive disease	4 (3.9%)

### No correlation was observed between molecular docking and the drug response

Drug sensitivity prediction with molecular docking focuses on somatic mutations in exons 18–21 of the EGFR tyrosine kinase domain and is based on the hypothesis that the docking score can serve as a metric for drug sensitivity. We calculated the docking scores for six approved drugs against 64 mutants and calculated their correlations with the experimental values. However, the calculated docking scores were not correlated with the experimental values (Maximal correlation *R*^2^ = 0.143; Figure S1), indicating that molecular docking may be an unreliable method for drug sensitivity prediction. The poor results may be due to the low accuracy of homology modeling, which cannot accurately reflect the protein structural changes caused by the residue mutation.

### Deep learning models with high prediction accuracy

**Figure 4:**
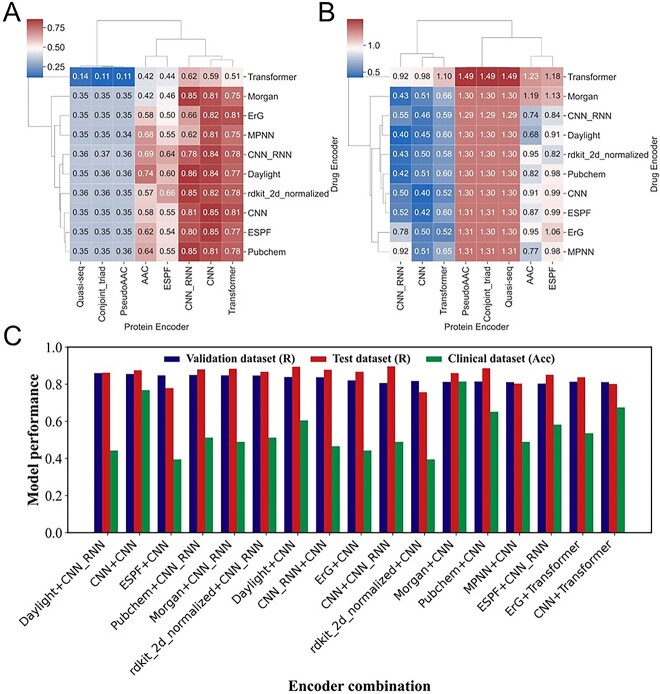
Evaluation of the deep learning models. (**A**) Heatmap of the Pearson correlation for 80 models. (**B**) Heatmap of mean squared error (MSE) for 80 models. (**C**) Model performance on the biological activity validation set, biological activity test set and representative D3EGFRdb subset.

The correlations between the scores predicted by 80 deep learning models and the experimental values were calculated. There were 17 models showing an average correlation >0.8, demonstrating the effectiveness of deep learning models in predicting the binding affinity of protein mutants and EGFR-TKIs (Figure 4**A** and **B**). For these 17 models, we merged the training and validation sets for retraining and re-evaluated their correlation with the test set. The results showed that 14 models had a correlation of >0.8 in the test set (Figure 4**C**). Furthermore, a multinomial logistic regression model was applied to map the predicted value with the drug response based on the representative D3EGFRdb subset. Finally, the Morgan + CNN model had the best performance, with correlation coefficients of 0.81 in the biological activity validation dataset and 0.86 in the biological activity test dataset, and its prediction accuracy in the representative D3EGFRdb subset was 0.81 (Table S4). Therefore, it was used as the final model for D3EGFRAI.

**Figure 5:**
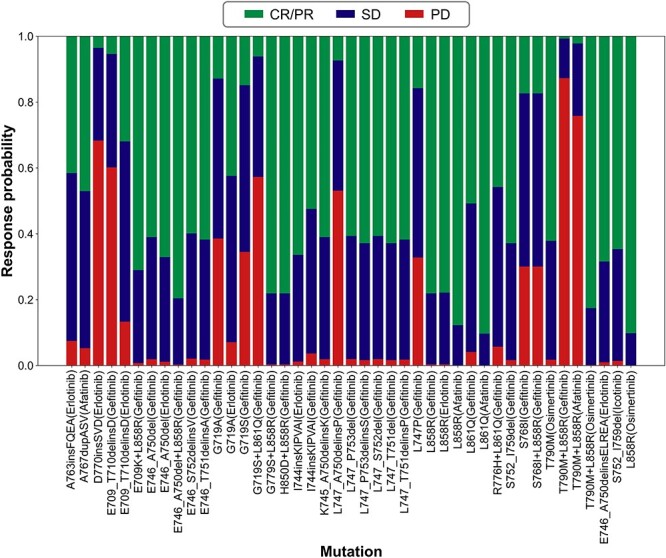
Predicted probability of each drug response for drug–mutant pairs in the representative D3EGFRdb subset.

As mentioned above, there may be one or two main drug responses with a higher probability in the same drug–mutant pair. Figure 5 shows the predicted probability of each drug response for drug–mutant pairs in the representative D3EGFRdb subset calculated by the predict_proba function of the logistic regression model. For example, the predicted probabilities of CR/PR, SD and PD of Afatinib-A767dupACS were 47.0%, 47.75% and 5.3%, respectively, indicating that both CR/PR and SD are the most likely drug responses for this drug–mutant pair. Therefore, taking only the drug response with the highest probability as the output cannot provide comprehensive information from a computational perspective. Therefore, the prediction results displayed by D3EGFRAI are both the predicted most likely drug response and the associated probabilities of each drug response. By re-evaluating the top two most likely drug responses predicted by the D3EGFRAI, the prediction accuracy improved from 0.81 to 0.95 for the representative D3EGFRdb subset. Finally, the D3EGFRAI model was applied to the external clinical dataset, in which the accuracy based on the drug response with the highest probability was 0.85 and that based on the top two drug responses with the highest probability was 0.92 (Table 3). In the external clinical dataset, 61.5% of drug–mutant pairs are not in the D3EGFRdb database, indicating that D3EGFRAI successfully maps binding affinity scores with drug response categories, thereby demonstrating its excellent generalization ability.

**Table 3:** ACR and predicted probability of each drug response on the external clinical dataset

Mutation	EGFR-TKI	ACR	P_CR/PR_	P_SD_	P_PD_
Ex19del	Osimertinib	CR/PR	99.0%	1.0%	0.0%
Ex19del	Icotinib	CR/PR	90.6%	9.4%	0.0%
Ex19del	Gefitinib	CR/PR	94.6%	5.4%	0.0%
L858R	Gefitinib	CR/PR	78.1%	21.6%	0.3%
Ex19del + T790M	Osimertinib	CR/PR	90.1%	9.9%	0.0%
L858R	Osimertinib	PD	90.2%	9.8%	0.0%
L858R	Erlotinib	CR/PR	77.8%	21.8%	0.4%
Ex19del	Erlotinib	SD	87.3%	12.7%	0.1%
L861Q	Gefitinib	CR/PR	50.8%	45.1%	4.1%
L858R	Icotinib	CR/PR	82.8%	17.1%	0.2%
Ex19del	Afatinib	CR/PR	90.2%	9.8%	0.0%
T790M+ L858R	Osimertinib	CR/PR	82.5%	17.3%	0.2%
S768I + L858R	Icotinib	CR/PR	71.3%	28.0%	0.7%

### Mutation scanning of key residues in the drug-binding pocket of EGFR using D3EGFRAI

Notably, amino acid mutations in the drug-binding site of EGFR can directly affect protein–drug-binding affinity. In this section, we focused on predicting the effect of potential mutations on drug sensitivity using D3EGFRAI. Nineteen crystal complex structures of approved drugs in the RCSB PDB database [51], including 1M17, 2ITO, 2ITY, 2ITZ, 3UG2, 4G5J, 4G5P, 4HJO, 4I22, 4I23, 4I24, 4WKQ, 4ZAU, 6JWL, 6JX0, 6JX4, 6JXT and 6LUD, were collected. A total of 26 residues were found within 4 Å around the protein pocket based on the reference ligands: L718, G719, S720, F723, V726, K728, A743, I744, K745, E762, M766, L788, T790, Q791, L792, M793, P794, F795, G796, C797, D800, E804, R841, L844, T854 and D855. By mutating these 26 residues into other 19 standard amino acids, 520 EGFR sequences were obtained for mutation affinity scanning, among which only 14 mutations (L718P, G719A, G719R, G719D, G719C, G719S, S720P, F723L, V726M, A743T, I744M, I744V, T790M and G796S) were reported previously. Figure S2 shows that mutations, including G796, T790, L718, L792, G719 and M766 residues, would significantly reduce the efficacy of first-generation EGFR–TKIs (e.g. erlotinib, gefitinib and icotinib), indicating high risks of potential new mutations to reduce the sensitivity of the drugs currently used. The second-generation drugs, afatinib and dacomitinib, have stronger binding affinity to most point mutations than the first- and third-generation drugs. This conclusion is consistent with the biological activity reported by Yasuda [31] and Robichaux [40], suggesting that severe adverse reactions may be related to excessive binding affinity [47, 52]. Third-generation drugs (olmutinib, osimertinib, almonertinib and furmonertinib) have limited effects on mutations in the C797, G796 and L718 residues, whose affinities for most mutations are generally stronger than those of first-generation drugs and weaker than those of second-generation drugs. Besides the point mutations mentioned above, there are various more complex mutations worth further exploration.

### D3EGFR input and output

For convenience, the D3EGFR server was constructed by integrating D3EGFRdb and D3EGFRAI for users to retrieve the collected drug response information and to predict drug response for rare and new mutations. Users can combine these two methods to determine the optimal drug treatment. The webserver supports English and Chinese (Simplified). D3EGFR is free for all users and no login is required.

**Figure 6:**
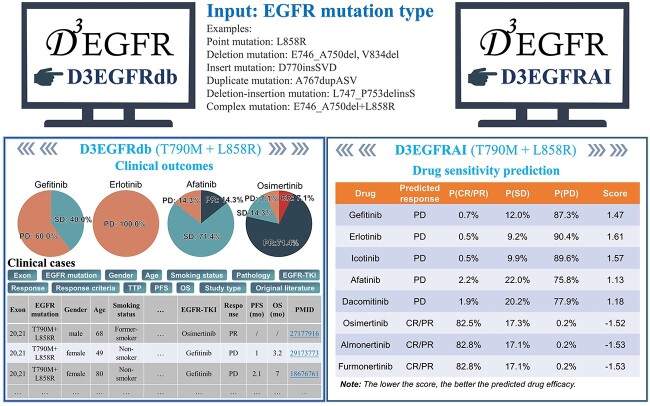
Input and output of the D3EGFR server.

Figure 6 shows the brief interfaces of the D3EGFR input and output. Noteworthy, the Ministry of Food and Drug Safety has prohibited doctors from prescribing olmutinib to new patients. Therefore, we removed olmutinib from the approved drug list in the prediction. The drug response retrieval in D3EGFRdb provided the statistical results of the drug response ratios of the mutants and drugs, as well as the specific clinical characteristics and original literature of each patient case. Taking the mutation T790M + L858R as an example, there were 29 patient cases in D3EGFRdb, in which the CP/PR response rate of osimertinib was 78.5%, superior to gefitinib (0%), erlotinib (0%) and afatinib (14.3%), indicating that osimertinib is an effective drug for treating patients with the T790M + L858R mutation. In addition, the predicted result of D3EGFRAI shows that the T790M + L858R mutation is sensitive to osimertinib and resistant to gefitinib, erlotinib and afatinib, consistent with D3EGFRdb results and previous reports [53]. In D3EGFRAI, users can obtain prediction results within 10 s by submitting a new mutation type.

## DISCUSSION

Drug sensitivity changes caused by protein mutations have seriously affected the therapeutic benefits of targeted drugs. There are hundreds of clinically reported EGFR mutations that have inconsistent drug responses primarily because of mutation-induced changes in protein–drug-binding affinity. At the atomic level, mutated residues may increase steric hindrance effects or influence interactions between the protein and the ligand, thereby causing changes in the drug’s binding ability and affecting the effectiveness of drug treatment.

As described in previous studies [54–56], there is increasing evidence that EGFR mutants can be used as predictive biomarkers for drug response in NSCLC. Therefore, this study selected EGFR mutation and drug response as variables to build a prediction model. Unlike previous studies [31, 32, 57–60], we manually collected large-scale patient cases from the literature over the past two decades to perform data-driven drug response prediction for all approved EGFR-TKIs. However, among the patient cases that were collected, it was found that patients with the same mutation may have different responses to the same drug treatment. This may be related to the patients’ individual characteristics and other factors. The specific reasons remain unclear. Therefore, we are collecting more data to introduce more variables in future model construction to enhance the model’s prediction performance.

## CONCLUSION

In this study, we developed the D3EGFR platform as a clinical-level drug recommendation tool to promote the development of precision medicine. Specifically, D3EGFRdb can provide real patient cases with specific clinical information and medication outcomes for convenient query, whereas D3EGFRAI is a drug response prediction model that has satisfactory prediction performance in clinical patient cases. Both methods will be useful in future clinical applications and scientific research. Based on real patient cases and prediction results provided by D3EGFR, clinicians can further combine their clinical experience and medical tests to decide on a more reasonable method of medication. More reported and internal clinical trial results in the future will be helpful to further improve the prediction accuracy and reliability of D3EGFR.

**Key Points**

- D3EGFR can efficiently retrieve drug responses based on D3EGFRdb.
- D3EGFR can make reliable drug response predictions based on D3EGFRAI.
- Mutation scanning of crucial residues for approved drugs was performed.

## FUNDING

This work was supported by the National Key Research and Development Program of China (2022YFA1004304), the National Natural Science Foundation of China (82322067, 82172991), the Natural Science Research Program for Higher Education in Jiangsu Province (21KJB320015), the Shanghai Health Commission (2019SY072) and the Shanghai Pulmonary Hospital Research Fund (FK18001, FKGG1805).

## DATA AVAILABILITY

The D3EGFR server is accessible freely at https://www.d3pharma.com/D3EGFR/index.php. The source code and dataset can be obtained from GitHub (https://github.com/Zhijian-Xu/D3EGFR) and zenodo (https://zenodo.org/records/10613332).

## Supplementary Material

D3EGFR-SI_bbae121

## Author Biographies

**Yulong Shi** is a PhD student at Shanghai Institute of Materia Medica. His research interests include computational biology and artificial intelligence.

**Chongwu Li** is a PhD student at Shanghai Pulmonary Hospital. His research interests include artificial intelligence-based precision medicine.

**Xinben Zhang** got his master’s degree at East China University of Science and Technology. His research interest is software development.

**Cheng Peng** got his PhD degree at Shanghai Institute of Materia Medica. His research interests include computer-aided drug design and molecular dynamics simulation.

**Peng Sun** is a master supervisor at the Nanjing Medical University. His research interests include biochemistry and molecular biology.

**Qian Zhang** is a master supervisor at the East China Normal University. Her research interests include machine learning and artificial intelligence.

**Leilei Wu** is a PhD student at Shanghai Pulmonary Hospital. His research interests include artificial intelligence-based precision medicine.

**Ying Ding** is a clinician at the First Affiliated Hospital of Nanjing Medical University. She is mainly engaged in precision medicine of the lung cancer.

**Dong Xie** is a clinician at the Shanghai Pulmonary Hospital. He is mainly engaged in precision surgical treatment of the early-stage lung cancer.

**Zhijian Xu** got his PhD degree at Shanghai Institute of Materia Medica in 2012. His research interests include drug-target interaction and virtual screening.

**Weiliang Zhu** received his PhD degree from Shanghai Institute of Materia Medica in 1998. His main research fields include computational biology, computational chemistry and pharmaceutical chemistry.

## References

[1] Sung H , Ferlay J, Siegel RL, et al. Global cancer statistics 2020: GLOBOCAN estimates of incidence and mortality worldwide for 36 cancers in 185 countries. CA Cancer J Clin 2021;71:209–49. 33538338 10.3322/caac.21660

[2] Siegel RL , Miller KD, Jemal A. Cancer statistics, 2016. CA Cancer J Clin 2016;66:7–30. 26742998 10.3322/caac.21332

[3] Lu T , Yang X, Huang Y, et al. Trends in the incidence, treatment, and survival of patients with lung cancer in the last four decades. Cancer Manag Res 2019;11:943–53. 30718965 10.2147/CMAR.S187317 PMC6345192

[4] Kosaka T , Yatabe Y, Endoh H, et al. Mutations of the epidermal growth factor receptor gene in lung cancer: biological and clinical implications. Cancer Res 2004;64:8919–23. 15604253 10.1158/0008-5472.CAN-04-2818

[5] Zhang YL , Yuan JQ, Wang KF, et al. The prevalence of EGFR mutation in patients with non-small cell lung cancer: a systematic review and meta-analysis. Oncotarget 2016;7:78985–93. 27738317 10.18632/oncotarget.12587 PMC5346692

[6] Sharma SV , Bell DW, Settleman J, Haber DA. Epidermal growth factor receptor mutations in lung cancer. Nat Rev Cancer 2007;7:169–81. 17318210 10.1038/nrc2088

[7] Red Brewer M , Yun CH, Lai D, et al. Mechanism for activation of mutated epidermal growth factor receptors in lung cancer. Proc Natl Acad Sci U S A 2013;110:E3595–604. 24019492 10.1073/pnas.1220050110 PMC3780914

[8] Vestergaard HH , Christensen MR, Lassen UN. A systematic review of targeted agents for non-small cell lung cancer. Acta Oncol 2018;57:176–86. 29172833 10.1080/0284186X.2017.1404634

[9] Mok TS , Wu YL, Thongprasert S, et al. Gefitinib or carboplatin-paclitaxel in pulmonary adenocarcinoma. N Engl J Med 2009;361:947–57. 19692680 10.1056/NEJMoa0810699

[10] Zhou C , Wu YL, Chen G, et al. Erlotinib versus chemotherapy as first-line treatment for patients with advanced EGFR mutation-positive non-small-cell lung cancer (OPTIMAL, CTONG-0802): a multicentre, open-label, randomised, phase 3 study. Lancet Oncol 2011;12:735–42. 21783417 10.1016/S1470-2045(11)70184-X

[11] Maemondo M , Inoue A, Kobayashi K, et al. Gefitinib or chemotherapy for non-small-cell lung cancer with mutated EGFR. N Engl J Med 2010;362:2380–8. 20573926 10.1056/NEJMoa0909530

[12] Mitsudomi T , Morita S, Yatabe Y, et al. Gefitinib versus cisplatin plus docetaxel in patients with non-small-cell lung cancer harbouring mutations of the epidermal growth factor receptor (WJTOG3405): an open label, randomised phase 3 trial. Lancet Oncol 2010;11:121–8. 20022809 10.1016/S1470-2045(09)70364-X

[13] Yu HA , Arcila ME, Rekhtman N, et al. Analysis of tumor specimens at the time of acquired resistance to EGFR-TKI therapy in 155 patients with EGFR-mutant lung cancers. Clin Cancer Res 2013;19:2240–7. 23470965 10.1158/1078-0432.CCR-12-2246 PMC3630270

[14] Kohsaka S , Nagano M, Ueno T, et al. A method of high-throughput functional evaluation of EGFR gene variants of unknown significance in cancer. Sci Transl Med 2017;9:eaan6566. 29141884 10.1126/scitranslmed.aan6566

[15] Kris MG , Johnson BE, Berry LD, et al. Using multiplexed assays of oncogenic drivers in lung cancers to select targeted drugs. JAMA 2014;311:1998–2006. 24846037 10.1001/jama.2014.3741 PMC4163053

[16] Tu HY , Ke EE, Yang JJ, et al. A comprehensive review of uncommon EGFR mutations in patients with non-small cell lung cancer. Lung Cancer 2017;114:96–102. 29173773 10.1016/j.lungcan.2017.11.005

[17] Sutiman N , Tan SW, Tan EH, et al. EGFR mutation subtypes influence survival outcomes following first-line gefitinib therapy in advanced Asian NSCLC patients. J Thorac Oncol 2017;12:529–38. 27908825 10.1016/j.jtho.2016.11.2225

[18] Park K , Yu CJ, Kim SW, et al. First-line erlotinib therapy until and beyond response evaluation criteria in solid tumors progression in Asian patients with epidermal growth factor receptor mutation-positive non-small-cell lung cancer: the ASPIRATION study. JAMA Oncol 2016;2:305–12. 26720423 10.1001/jamaoncol.2015.4921

[19] Remon J , Caramella C, Jovelet C, et al. Osimertinib benefit in EGFR-mutant NSCLC patients with T790M-mutation detected by circulating tumour DNA. Ann Oncol 2017;28:784–90. 28104619 10.1093/annonc/mdx017

[20] Lee J , Choi Y, Han J, et al. Osimertinib improves overall survival in patients with EGFR-mutated NSCLC with leptomeningeal metastases regardless of T790M mutational status. J Thorac Oncol 2020;15:1758–66. 32652216 10.1016/j.jtho.2020.06.018

[21] Shigematsu H , Lin L, Takahashi T, et al. Clinical and biological features associated with epidermal growth factor receptor gene mutations in lung cancers. J Natl Cancer Inst 2005;97:339–46. 15741570 10.1093/jnci/dji055

[22] Patterson S , Statz C, Yin T, Mockus S. The JAX clinical knowledgebase: a valuable resource for identifying evidence related to complex molecular signatures in different types of cancer. Cancer Genet 2017;214-215:33.

[23] Griffith M , Spies NC, Krysiak K, et al. CIViC is a community knowledgebase for expert crowdsourcing the clinical interpretation of variants in cancer. Nat Genet 2017;49:170–4. 28138153 10.1038/ng.3774 PMC5367263

[24] Huang L , Fernandes H, Zia H, et al. The cancer precision medicine knowledge base for structured clinical-grade mutations and interpretations. J Am Med Inform Assoc 2017;24:513–9. 27789569 10.1093/jamia/ocw148 PMC5391733

[25] Bamford S , Dawson E, Forbes S, et al. The COSMIC (catalogue of somatic mutations in cancer) database and website. Br J Cancer 2004;91:355–8. 15188009 10.1038/sj.bjc.6601894 PMC2409828

[26] Chakravarty D , Gao J, Phillips SM, et al. OncoKB: a precision oncology knowledge base. JCO Precis Oncol 2017;1:1–16. 10.1200/PO.17.00011 PMC5586540 28890946

[27] Swanton C . My cancer genome: a unified genomics and clinical trial portal. Lancet Oncol 2012;13:668–9.22748256

[28] Chou TY , Chiu CH, Li LH, et al. Mutation in the tyrosine kinase domain of epidermal growth factor receptor is a predictive and prognostic factor for gefitinib treatment in patients with non-small cell lung cancer. Clin Cancer Res 2005;11:3750–7. 15897572 10.1158/1078-0432.CCR-04-1981

[29] Fang S , Wang Z. EGFR mutations as a prognostic and predictive marker in non-small-cell lung cancer. Drug Des Dev Ther 2014;8:1595–611. 10.2147/DDDT.S69690 PMC4189714 25302015

[30] Han SW , Kim TY, Hwang PG, et al. Predictive and prognostic impact of epidermal growth factor receptor mutation in non-small-cell lung cancer patients treated with gefitinib. J Clin Oncol 2005;23:2493–501. 15710947 10.1200/JCO.2005.01.388

[31] Ikemura S , Yasuda H, Matsumoto S, et al. Molecular dynamics simulation-guided drug sensitivity prediction for lung cancer with rare EGFR mutations. Proc Natl Acad Sci U S A 2019;116:10025–30. 31043566 10.1073/pnas.1819430116 PMC6525482

[32] Wang DD , Zhou W, Yan H, et al. Personalized prediction of EGFR mutation-induced drug resistance in lung cancer. Sci Rep 2013;3:2855. 24092472 10.1038/srep02855 PMC3790204

[33] Öztürk H , Özgür A, Ozkirimli E. DeepDTA: deep drug-target binding affinity prediction. Bioinformatics 2018;34:i821–9. 30423097 10.1093/bioinformatics/bty593 PMC6129291

[34] Yang Y , Zhou D, Zhang X, et al. D3AI-CoV: a deep learning platform for predicting drug targets and for virtual screening against COVID-19. Brief Bioinform 2022;23:bbac147. 10.1093/bib/bbac147 PMC9310271 35443040

[35] Wheeler DL , Church DM, Edgar R, et al. Database resources of the National Center for biotechnology information: update. Nucleic Acids Res 2004;32:35D–40. 14681353 10.1093/nar/gkh073 PMC308807

[36] Miller AB , Hoogstraten B, Staquet M, Winkler A. Reporting results of cancer treatment. Cancer 1981;47:207–14. 7459811 10.1002/1097-0142(19810101)47:1<207::aid-cncr2820470134>3.0.co;2-6

[37] Therasse P , Arbuck SG, Eisenhauer EA, et al. New guidelines to evaluate the response to treatment in solid tumors. European Organization for Research and Treatment of cancer, National Cancer Institute of the United States, National Cancer Institute of Canada. J Natl Cancer Inst 2000;92:205–16. 10655437 10.1093/jnci/92.3.205

[38] Eisenhauer EA , Therasse P, Bogaerts J, et al. New response evaluation criteria in solid tumours: revised RECIST guideline (version 1.1). Eur J Cancer 2009;45:228–47. 19097774 10.1016/j.ejca.2008.10.026

[39] Kitchen DB , Decornez H, Furr JR, Bajorath J. Docking and scoring in virtual screening for drug discovery: methods and applications. Nat Rev Drug Discov 2004;3:935–49. 15520816 10.1038/nrd1549

[40] Robichaux JP , Le X, Vijayan RSK, et al. Structure-based classification predicts drug response in EGFR-mutant NSCLC. Nature 2021;597:732–7. 34526717 10.1038/s41586-021-03898-1 PMC8481125

[41] Webb B , Sali A. Comparative protein structure modeling using MODELLER. Curr Protoc Bioinformatics 2016;54:1–5. 10.1002/cpbi.3 PMC5031415 27322406

[42] Dolinsky TJ , Nielsen JE, McCammon JA, Baker NA. PDB2PQR: an automated pipeline for the setup of Poisson-Boltzmann electrostatics calculations. Nucleic Acids Res 2004;32:W665–7. 15215472 10.1093/nar/gkh381 PMC441519

[43] Koes DR , Baumgartner MP, Camacho CJ. Lessons learned in empirical scoring with smina from the CSAR 2011 benchmarking exercise. J Chem Inf Model 2013;53:1893–904. 23379370 10.1021/ci300604z PMC3726561

[44] Trott O , Olson AJ. AutoDock Vina: improving the speed and accuracy of docking with a new scoring function, efficient optimization, and multithreading. J Comput Chem 2010;31:455–61. 19499576 10.1002/jcc.21334 PMC3041641

[45] LeCun Y , Bengio Y, Hinton G. Deep learning. Nature 2015;521:436–44. 26017442 10.1038/nature14539

[46] Huang K , Fu T, Glass LM, et al. DeepPurpose: a deep learning library for drug-target interaction prediction. Bioinformatics 2021;36:5545–7. 33275143 10.1093/bioinformatics/btaa1005 PMC8016467

[47] Takeda M , Okamoto I, Nakagawa K. Pooled safety analysis of EGFR-TKI treatment for EGFR mutation-positive non-small cell lung cancer. Lung Cancer 2015;88:74–9. 25704957 10.1016/j.lungcan.2015.01.026

[48] Ramalingam SS , O'Byrne K, Boyer M, et al. Dacomitinib versus erlotinib in patients with EGFR-mutated advanced nonsmall-cell lung cancer (NSCLC): pooled subset analyses from two randomized trials. Ann Oncol 2016;27:423–9. 26768165 10.1093/annonc/mdv593

[49] Remon J , Steuer CE, Ramalingam SS, Felip E. Osimertinib and other third-generation EGFR TKI in EGFR-mutant NSCLC patients. Ann Oncol 2018;29:i20–7. 29462255 10.1093/annonc/mdx704

[50] Chen X , Zhu Q, Liu Y, et al. Icotinib is an active treatment of non-small-cell lung cancer: a retrospective study. PloS One 2014;9:e95897. 24836053 10.1371/journal.pone.0095897 PMC4023939

[51] Burley SK , Berman HM, Bhikadiya C, et al. RCSB protein data Bank: biological macromolecular structures enabling research and education in fundamental biology, biomedicine, biotechnology and energy. Nucleic Acids Res 2019;47:D464–74. 30357411 10.1093/nar/gky1004 PMC6324064

[52] Wu YL , Cheng Y, Zhou X, et al. Dacomitinib versus gefitinib as first-line treatment for patients with EGFR-mutation-positive non-small-cell lung cancer (ARCHER 1050): a randomised, open-label, phase 3 trial. Lancet Oncol 2017;18:1454–66. 28958502 10.1016/S1470-2045(17)30608-3

[53] Hsu WH , Yang JC, Mok TS, Loong HH. Overview of current systemic management of EGFR-mutant NSCLC. Ann Oncol 2018;29:i3–9. 29462253 10.1093/annonc/mdx702

[54] Del Re M , Rofi E, Cappelli C, et al. The increase in activating EGFR mutation in plasma is an early biomarker to monitor response to osimertinib: a case report. BMC Cancer 2019;19:410. 31039766 10.1186/s12885-019-5604-6 PMC6492432

[55] Kaneko K , Kumekawa Y, Makino R, et al. EGFR gene alterations as a prognostic biomarker in advanced esophageal squamous cell carcinoma. Front Biosci (Landmark Ed) 2010;15:65–72. 20036807 10.2741/3607

[56] Dahabreh IJ , Linardou H, Siannis F, et al. Somatic EGFR mutation and gene copy gain as predictive biomarkers for response to tyrosine kinase inhibitors in non–small cell lung cancer. Clin Cancer Res 2010;16:291–303. 20028749 10.1158/1078-0432.CCR-09-1660

[57] Zou B , Lee VHF, Yan H. Prediction of sensitivity to gefitinib/erlotinib for EGFR mutations in NSCLC based on structural interaction fingerprints and multilinear principal component analysis. BMC Bioinformatics 2018;19:1–13. 29514601 10.1186/s12859-018-2093-6 PMC5842518

[58] Wang DD , Lee VHF, Zhu G, et al. Selectivity profile of afatinib for EGFR-mutated non-small-cell lung cancer. Mol Biosyst 2016;12(5):1552–63. 26961138 10.1039/c6mb00038j

[59] Chiu YC , Chen HIH, Zhang T, et al. Predicting drug response of tumors from integrated genomic profiles by deep neural networks. BMC Med Genomics 2019;12(S1):143–55. 30704458 10.1186/s12920-018-0460-9 PMC6357352

[60] Ma L , Wang DD, Zou B, Yan H. An eigen-binding site based method for the analysis of anti-EGFR drug resistance in lung cancer treatment. IEEE/ACM Trans Comput Biol Bioinform 2016;14(5):1187–94. 27187970 10.1109/TCBB.2016.2568184

