# Supplementary material for: D3EGFR: a webserver for deep learning-guided drug sensitivity prediction and drug response information retrieval for EGFR mutation-driven lung cancer
Source: Brief Bioinform. 2024 Mar 28;25(3):bbae121. doi: 10.1093/bib/bbae121 (PMC10981678; doi:10.1093/bib/bbae121)
Supplement: D3EGFR-SI_bbae121 [file d3egfr-si_bbae121.docx]

**Supplementary Information**

**D3EGFR: a webserver for deep learning-guided drug sensitivity prediction and drug response information retrieval for EGFR mutation-driven lung cancer**

Yulong Shi^a,b,†^, Chongwu Li^c,†^, Xinben Zhang^a,†^, Cheng Peng^a,b^, Peng Sun^d^, Qian Zhang^e^, Leilei Wu^c^, Ying Ding^f,^*^*^*, Dong Xie^c,*^, Zhijian Xu^a,b,^*^*^*, Weiliang Zhu^a,b,^*^*^*

^a^State Key Laboratory of Drug Research; Drug Discovery and Design Center, Shanghai Institute of Materia Medica, Chinese Academy of Sciences, Shanghai 201203, China

^b^School of Pharmacy, University of Chinese Academy of Sciences, Beijing 100049, China

^c^Department of Thoracic Surgery, Shanghai Pulmonary Hospital, Tongji University School of Medicine, Shanghai 200433, China

^d^Key Laboratory of Human Functional Genomics of Jiangsu Province, Department of Biochemistry and Molecular Biology, Nanjing Medical University, Nanjing 211166, China

^e^School of Computer Science and Technology, East China Normal University, Shanghai 200062, China

^f^Department of Pathology, the First Affiliated Hospital of Nanjing Medical University, Nanjing 210029, China

^*^Corresponding authors: Ying Ding, Department of Pathology, the First Affiliated Hospital of Nanjing Medical University, Nanjing 210029, China. Tel.: +86-25-68303714. E-mail: dingying@njmu.edu.cn; Dong Xie, Department of Thoracic Surgery, Shanghai Pulmonary Hospital, Tongji University School of Medicine, Shanghai 200433, China. TEL.: +86-21-65115006. E-mail: kongduxd@163.com; Zhijian Xu, State Key Laboratory of Drug Research; Drug Discovery and Design Center, Shanghai Institute of Materia Medica, Chinese Academy of Sciences, Shanghai 201203, China; School of Pharmacy, University of Chinese Academy of Sciences, Beijing 100049, China. Tel.: +86-21-68077841. E-mail: zjxu@simm.ac.cn; Weiliang Zhu, State Key Laboratory of Drug Research; Drug Discovery and Design Center, Shanghai Institute of Materia Medica, Chinese Academy of Sciences, Shanghai 201203, China; School of Pharmacy, University of Chinese Academy of Sciences, Beijing 100049, China. Tel.: +86-21-50805020. E-mail: wlzhu@simm.ac.cn

^†^These authors contributed equally to this work.

**Table S1** Approved small molecule drugs indicated for EGFR mutation-positive NSCLC.

| No. | Generic Name | Brand Name | Company | Launch year | First country |
| --- | --- | --- | --- | --- | --- |
| 1 | Gefitinib | Iressa | AstraZeneca | 2003 | Japan |
| 2 | Erlotinib | Tarceva | Roche Pharmaceuticals | 2004 | America |
| 3 | Icotinib | Conmana | Betta Pharmaceuticals | 2011 | China |
| 4 | Afatinib | Gilotrif; Tomtovok; Tovok | Boehringer Ingelheim | 2013 | America |
| 5 | Osimertinib | Tagrisso | AstraZeneca | 2015 | America |
| 6 | Olmutinib | Olita | Hanmi Pharmaceuticals | 2016 | Korea |
| 7 | Dacomitinib | Vizimpro | Pfizer | 2018 | America |
| 8 | Almonertinib | Ameile; Amerol | Jiangsu Hansoh Pharmaceutical | 2020 | China |
| 9 | Furmonertinib | Ivesa | Allist Pharmaceuticals | 2021 | China |

**Table S2** Related drug sensitivity prediction studies for EGFR mutations.

| Reference No. | Method | Predicted drug | Predicted value |
| --- | --- | --- | --- |
| [1] | MD and multilinear principal component analysis | gefitinib and erlotinib | IC_50_ in vitro |
| [2] | MD | afatinib | drug response at clinical level |
| [3] | MD | osimertinib | drug sensitivity of exon 20 insertion mutants in vitro and in vivo |
| [4] | MD and extreme learning machine | gefinitib and erlotinib | drug response and survival time at clinical level |
| [5] | Deep Learning | afatinib and gefitinib | IC_50_ in vitro |
| [6] | MD and SVM | gefitinib | drug response at clinical level |

**Table S3** Clinical characteristics and EGFR mutation status of 102 NSCLC patients.

| No. | Patient | Smoking status | Pathology | Type | EGFR-TKI | Response | Survival status | PFS (mo) | OS (mo) |
| --- | --- | --- | --- | --- | --- | --- | --- | --- | --- |
| 1 | M/63 | Non | ADC | Ex19del | Osimertinib | PR | Alive | 10 | 12 |
| 2 | M/85 | Former | ADC | Ex19del | Icotinib | PR | Alive | 2 | 2 |
| 3 | M/72 | Former | ADC | Ex19del | Gefitinib | SD | Alive | 24 | 24 |
| 4 | F/68 | Non | ADC | Ex19del | Icotinib | PR | Alive | 11 | 24 |
| 5 | F/56 | Non | ADC | Ex19del | Gefitinib | PR | Alive | 32 | 32 |
| 6 | F/69 | Non | ADC | L858R | Gefitinib | SD | Alive | 2 | 4 |
| 7 | F/65 | Non | ADC | Ex19del | Gefitinib | PR | Alive | 20 | 20 |
| 8 | M/73 | Non | ADC | Ex19del | Gefitinib | SD | Alive | 12 | 12 |
| 9 | M/55 | Non | ADC | Ex19del | Gefitinib | PR | Alive | 9 | 20 |
| 10 | F/64 | Non | ADC | Ex19del+  T790M | Osimertinib | PR | Alive | 8 | 46 |
| 11 | F/66 | Non | ADC | L858R | Gefitinib | SD | Alive | 3 | 7 |
| 12 | F/56 | Non | LCC | L858R | Osimertinib | PD | Alive | 1 | 5 |
| 13 | F/68 | Non | ADC | L858R | Gefitinib | PR | Alive | 8 | 8 |
| 14 | M/62 | current | ADC | L858R | Erlotinib | PR | Dead | 15 | 30 |
| 15 | M/61 | Former | ADC | Ex19del | Erlotinib | SD | Alive | 13 | 37 |
| 16 | F/37 | Non | ADC | L858R | Gefitinib | PR | Dead | 9 | 27 |
| 17 | F/46 | Non | ADC | L858R | Gefitinib | PR | Alive | 18 | 33 |
| 18 | M/54 | Former | ADC | Ex19del | Erlotinib | SD | Dead | 4 | 22 |
| 19 | F/65 | Non | ADC | L861Q | Gefitinib | PR | Dead | 24 | 65 |
| 20 | F/50 | Non | ADC | Ex19del | Gefitinib | SD | Dead | 7 | 18 |
| 21 | M/53 | Non | ADC | Ex19del+  T790M | Osimertinib | PR | Dead | 22 | 65 |
| 22 | F/68 | Non | ADC | L858R | Gefitinib | PR | Dead | 7 | 27 |
| 23 | M/64 | Former | ADC | Ex19del | Gefitinib | PR | Dead | 4 | 30 |
| 24 | F/63 | Non | ADC | L858R | Gefitinib | PR | Alive | 33 | 36 |
| 25 | F/51 | Non | ADC | Ex19del | Gefitinib | PR | Dead | 17 | 45 |
| 26 | M/63 | Former | ADC | Ex19del | Icotinib | PD | Dead | 1 | 29 |
| 27 | F/58 | Non | ADC | L858R | Erlotinib | SD | Alive | 12 | 29 |
| 28 | M/65 | Non | ADC | Ex19del | Gefitinib | PR | Dead | 6 | 30 |
| 29 | M/68 | Non | ADC | L858R | Gefitinib | PR | Dead | 14 | 48 |
| 30 | F/53 | Non | ADC | L858R | Erlotinib | SD | Dead | 12 | 21 |
| 31 | M/61 | Non | ADC | Ex19del | Gefitinib | PR | Dead | 11 | 62 |
| 32 | M/33 | Former | ADC | Ex19del | Icotinib | PR | Alive | 4 | 36 |
| 33 | F/67 | Non | ADC | L861Q | Gefitinib | PR | Dead | 24 | 68 |
| 34 | F/68 | Non | ADC | L858R | Gefitinib | PR | Dead | 6 | 30 |
| 35 | F/54 | Non | ADC | L858R | Icotinib | SD | Dead | 4 | 20 |
| 36 | M/70 | current | ADC | Ex19del | Erlotinib | SD | Dead | 4 | 27 |
| 37 | F/55 | Non | ADC | Ex19del | Gefitinib | SD | Alive | 6 | 46 |
| 38 | M/49 | Non | ADC | Ex19del | Icotinib | PR | Dead | 5 | 28 |
| 39 | F/56 | Non | ADC | Ex19del | Icotinib | SD | Alive | 3 | 48 |
| 40 | M/48 | Non | ADC | Ex19del | Icotinib | SD | Dead | 4 | 18 |
| 41 | M/53 | Non | ADC | L858R | Erlotinib | PR | Dead | 3 | 28 |
| 42 | M/41 | Former | ADC | L858R | Gefitinib | PR | Alive | 13 | 38 |
| 43 | M/56 | Former | ADC | Ex19del | Icotinib | PR | Dead | 11 | 47 |
| 44 | M/69 | Non | ADC | Ex19del | Icotinib | PR | Dead | 16 | 43 |
| 45 | M/45 | Former | ADC | Ex19del | Afatinib | PR | Alive | 17 | 39 |
| 46 | M/67 | Former | ADC | Ex19del | Icotinib | PR | Dead | 8 | 48 |
| 47 | F/54 | Non | ADC | Ex19del | Icotinib | PR | Dead | 15 | 41 |
| 48 | F/55 | Non | ADC | Ex19del | Icotinib | PR | Alive | 12 | 46 |
| 49 | F/63 | Non | ADC | Ex19del | Gefitinib | PR | Dead | 9 | 34 |
| 50 | M/63 | Non | ADC | L858R | Icotinib | PR | Dead | 8 | 29 |
| 51 | F/54 | Non | ADC | L858R | Icotinib | SD | Dead | 4 | 22 |
| 52 | F/58 | Non | ADC | L858R | Icotinib | PR | Dead | 9 | 20 |
| 53 | F/55 | Non | ADC | Ex19del | Gefitinib | PR | Alive | 19 | 55 |
| 54 | F/78 | Non | ADC | Ex19del | Gefitinib | PR | Dead | 15 | 66 |
| 55 | M/58 | Former | ADC | L858R | Erlotinib | PR | Dead | 4 | 25 |
| 56 | F/75 | Non | ADC | L858R | Gefitinib | PR | Alive | 9 | 43 |
| 57 | F/55 | Non | ADC | L858R | Gefitinib | SD | Alive | 5 | 53 |
| 58 | F/70 | Non | ADC | Ex19del | Icotinib | PR | Alive | 13 | 37 |
| 59 | M/71 | Former | ADC | Ex19del | Gefitinib | SD | Alive | 8 | 34 |
| 60 | M/69 | current | ADC | Ex19del | Icotinib | SD | Dead | 3 | 12 |
| 61 | M/60 | Former | ADC | Ex19del | Gefitinib | PR | Alive | 8 | 36 |
| 62 | F/75 | Non | ADC | L858R | Icotinib | PR | Alive | 23 | 44 |
| 63 | M/51 | Former | ADC | L858R | Gefitinib | SD | Dead | 2 | 21 |
| 64 | M/61 | Former | ADC | Ex19del | Gefitinib | SD | Dead | 11 | 17 |
| 65 | F/54 | Non | ADC | L858R | Gefitinib | PR | Alive | 23 | 49 |
| 66 | M/59 | Former | ADC | L858R | Gefitinib | PR | Dead | 15 | 30 |
| 67 | M/39 | Non | ADC | L858R | Erlotinib | PR | Alive | 25 | 39 |
| 68 | M/63 | Non | ADC | L858R | Gefitinib | PR | Dead | 20 | 23 |
| 69 | M/67 | Former | ADC | L858R | Gefitinib | PR | Alive | 42 | 42 |
| 70 | M/62 | Non | ADC | Ex19del | Erlotinib | PR | Alive | 13 | 13 |
| 71 | M/64 | Former | ADC | Ex19del | Gefitinib | PR | Alive | 15 | 15 |
| 72 | F/48 | Non | SCC | Ex19del | Gefitinib | SD | Dead | 8 | 25 |
| 73 | F/72 | Non | ADC | Ex19del | Gefitinib | PR | Alive | 34 | 41 |
| 74 | M/35 | Non | ADC | Ex19del | Gefitinib | SD | Alive | 9 | 39 |
| 75 | F/60 | Non | ADC | L858R | Gefitinib | SD | Dead | 8 | 53 |
| 76 | M/48 | Former | ADC | Ex19del | Gefitinib | PR | Dead | 9 | 38 |
| 77 | F/55 | Non | ADC | L858R | Gefitinib | PR | Alive | 22 | 53 |
| 78 | F/69 | Non | ADC | L858R | Gefitinib | SD | Alive | 32 | 32 |
| 79 | M/54 | Former | ADC | Ex19del | Icotinib | SD | Dead | 4 | 21 |
| 80 | F/65 | Non | ADC | Ex19del | Icotinib | PR | Alive | 13 | 49 |
| 81 | M/60 | Former | ADC | L858R | Gefitinib | SD | Dead | 8 | 25 |
| 82 | F/70 | Non | ADC | Ex19del | Icotinib | PR | Alive | 16 | 32 |
| 83 | M/71 | Former | ADC | L858R | Gefitinib | PR | Dead | 9 | 27 |
| 84 | F/67 | Non | ADC | L858R | Gefitinib | PR | Alive | 7 | 34 |
| 85 | F/46 | Non | ADC | Ex19del | Icotinib | PR | Dead | 5 | 23 |
| 86 | M/50 | Non | ADC | Ex19del | Gefitinib | PR | Alive | 36 | 36 |
| 87 | F/65 | Non | ADC | L858R | Gefitinib | SD | Dead | 9 | 39 |
| 88 | F/71 | Non | ADC | L858R+  T790M | Osimertinib | PR | Alive | 31 | 31 |
| 89 | M/60 | Former | ADC | L858R | Icotinib | PR | Dead | 30 | 49 |
| 90 | F/48 | Non | ADC | Ex19del | Icotinib | SD | Dead | 6 | 45 |
| 91 | F/60 | Non | ADC | Ex19del | Gefitinib | PD | Dead | 1 | 13 |
| 92 | M/67 | Non | ADC | L858R | Icotinib | SD | Dead | 3 | 25 |
| 93 | M/76 | Former | ADC | S768I+  L858R | Icotinib | PR | Alive | 18 | 35 |
| 94 | F/50 | Non | ADC | Ex19del | Icotinib | SD | Alive | 24 | 36 |
| 95 | M/47 | Former | ADC | L858R | Icotinib | SD | Dead | 37 | 56 |
| 96 | M/55 | Former | ADC | Ex19del | Icotinib | PR | Dead | 11 | 37 |
| 97 | M/63 | Non | ADC | Ex19del | Gefitinib | PR | Dead | 8 | 35 |
| 98 | M/66 | Former | ADC | L858R | Icotinib | PR | Dead | 10 | 26 |
| 99 | F/70 | Non | ADC | L858R | Gefitinib | PR | Dead | 8 | 30 |
| 100 | F/52 | Non | ADC | Ex19del | Icotinib | PR | Alive | 11 | 31 |
| 101 | F/73 | Non | ADC | Ex19del | Icotinib | PD | Dead | 2 | 15 |
| 102 | F/66 | Non | ADC | L858R | Icotinib | PR | Alive | 8 | 49 |

Abbreviation: F, female; M, male; ADC, adenocarcinoma; LCC, large cell carcinoma; SCC, squamous cell carcinoma; PR, partial response; SD, stable disease; PD, progressive disease; mo, month.

**Table S4** The predicted responses of drug-mutant pairs in representative D3EGFRdb subset.

| No. | Mutation | EGFR-TKI | ACR | Predicted response | Score |
| --- | --- | --- | --- | --- | --- |
| 1 | A763insFQEA | Erlotinib | CR/PR | SD | -0.31 |
| 2 | A767dupASV | Afatinib | SD | SD | -0.44 |
| 3 | D770insSVD | Erlotinib | PD | PD | 0.97 |
| 4 | E709_T710delinsD | Gefitinib | PD | PD | 0.82 |
| 5 | E709_T710delinsD | Erlotinib | SD | SD | -0.08 |
| 6 | E709K+L858R | Gefitinib | CR/PR | CR/PR | -1.08 |
| 7 | E746_A750del | Gefitinib | CR/PR | CR/PR | -0.79 |
| 8 | E746_A750del | Erlotinib | CR/PR | CR/PR | -0.96 |
| 9 | E746_A750del+L858R | Gefitinib | CR/PR | CR/PR | -1.39 |
| 10 | E746_S752delinsV | Gefitinib | CR/PR | CR/PR | -0.76 |
| 11 | E746_T751delinsA | Gefitinib | CR/PR | CR/PR | -0.81 |
| 12 | G719A | Gefitinib | SD | SD | 0.46 |
| 13 | G719A | Erlotinib | SD | SD | -0.33 |
| 14 | G719S | Gefitinib | SD | SD | 0.39 |
| 15 | G719S+L861Q | Gefitinib | SD | PD | 0.77 |
| 16 | G779S+L858R | Gefitinib | CR/PR | CR/PR | -1.33 |
| 17 | H850D+L858R | Gefitinib | SD | CR/PR | -1.33 |
| 18 | I744insKIPVAI | Erlotinib | CR/PR | CR/PR | -0.94 |
| 19 | I744insKIPVAI | Gefitinib | SD | CR/PR | -0.57 |
| 20 | K745_A750delinsK | Gefitinib | CR/PR | CR/PR | -0.79 |
| 21 | L747_A750delinsP | Gefitinib | CR/PR | PD | 0.7 |
| 22 | L747_P753del | Gefitinib | CR/PR | CR/PR | -0.78 |
| 23 | L747_P753delinsS | Gefitinib | CR/PR | CR/PR | -0.84 |
| 24 | L747_S752del | Gefitinib | CR/PR | CR/PR | -0.78 |
| 25 | L747_T751del | Gefitinib | CR/PR | CR/PR | -0.84 |
| 26 | L747_T751delinsP | Gefitinib | CR/PR | CR/PR | -0.81 |
| 27 | L747P | Gefitinib | SD | SD | 0.36 |
| 28 | L858R | Gefitinib | CR/PR | CR/PR | -1.33 |
| 29 | L858R | Erlotinib | CR/PR | CR/PR | -1.32 |
| 30 | L858R | Afatinib | CR/PR | CR/PR | -1.8 |
| 31 | L861Q | Gefitinib | SD | CR/PR | -0.53 |
| 32 | L861Q | Afatinib | CR/PR | CR/PR | -1.98 |
| 33 | R776H+L861Q | Gefitinib | SD | SD | -0.41 |
| 34 | S752_I759del | Gefitinib | CR/PR | CR/PR | -0.84 |
| 35 | S768I | Gefitinib | SD | SD | 0.31 |
| 36 | S768I+L858R | Gefitinib | CR/PR | SD | 0.31 |
| 37 | T790M | Osimertinib | CR/PR | CR/PR | -0.82 |
| 38 | T790M+L858R | Gefitinib | PD | PD | 1.47 |
| 39 | T790M+L858R | Afatinib | SD | PD | 1.13 |
| 40 | T790M+L858R | Osimertinib | CR/PR | CR/PR | -1.52 |
| 41 | E746_A750delinsELREA | Erlotinib | CR/PR | CR/PR | -1 |
| 42 | S752_I759del | Icotinib | CR/PR | CR/PR | -0.89 |
| 43 | L858R | Osimertinib | CR/PR | CR/PR | -1.97 |

**
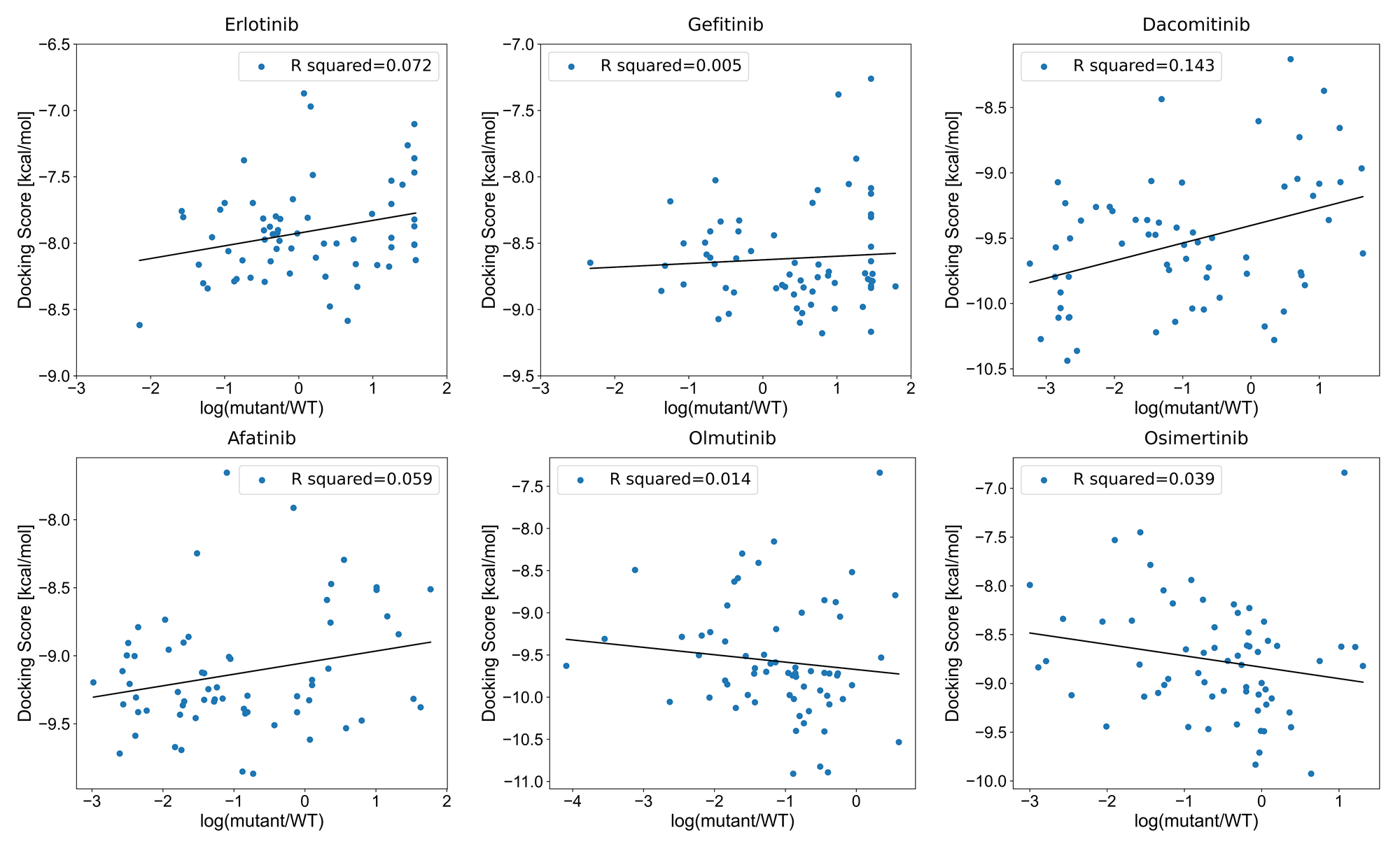
**

**Figure S1** Plot of docking scores against experimental values. Each dot represents an EGFR mutant and the line represents a linear fit with squared correlation coefficient R^2^.


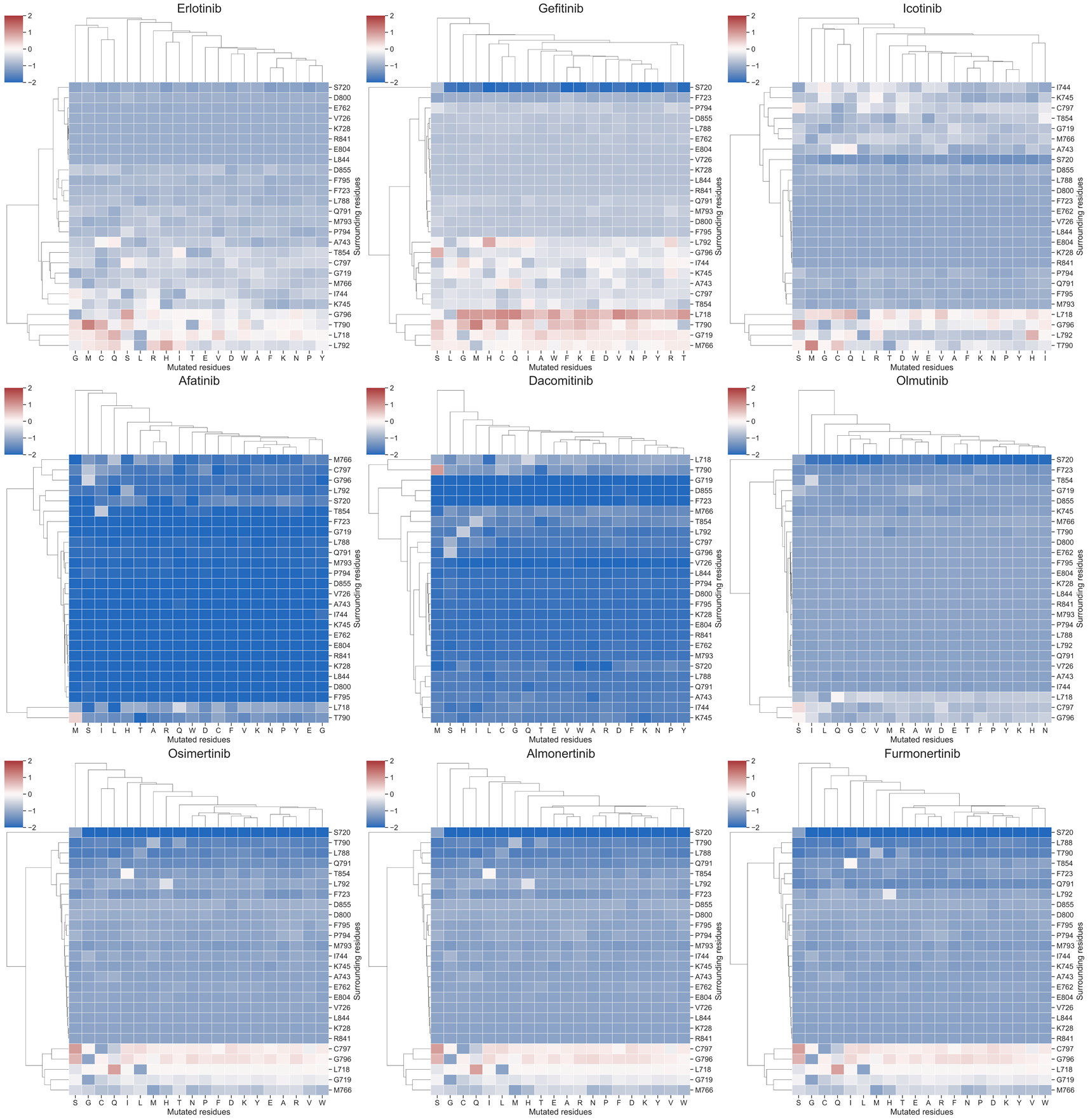


**Figure S2** Mutation affinity scanning of surrounding residues for approved EGFR-TKIs.

**Reference**

1. Zou B, Lee V H F, Yan H. Prediction of sensitivity to gefitinib/erlotinib for EGFR mutations in NSCLC based on structural interaction fingerprints and multilinear principal component analysis, BMC bioinformatics 2018;19:1-13.

2. Wang D D, Lee V H F, Zhu G, et al. Selectivity profile of afatinib for EGFR-mutated non-small-cell lung cancer, Mol Biosyst 2016;12(5):1552-1563.

3. Ikemura S, Yasuda H, Matsumoto S et al. Molecular dynamics simulation-guided drug sensitivity prediction for lung cancer with rare EGFR mutations, Proc Natl Acad Sci U S A 2019;116:10025-10030.

4. Wang DD, Zhou W, Yan H et al. Personalized prediction of EGFR mutation-induced drug resistance in lung cancer, Sci Rep 2013;3:2855.

5. Chiu Y C, Chen H I H, Zhang T, et al. Predicting drug response of tumors from integrated genomic profiles by deep neural networks, BMC Med Genomics 2019;12(1):143-155.

6. Ma L, Wang D D, Zou B, et al. An eigen-binding site based method for the analysis of anti-EGFR drug resistance in lung cancer treatment, IEEE/ACM Trans Comput Biol Bioinform 2016;14(5):1187-1194.
